# The Surgical Aspect of Abdominal Tuberculosis

**Published:** 1892-03-05

**Authors:** 


### SURGERY.

### THE SURGICAL ASPECT OF ABDOMINAL TUBERCULOSIS.

The surgeon at the present day is always making inroads into the province of the physician. He now removes cerebral tumours, and excises the apex of the lung. It is not to be wondered at then, that he should have encroached on the peritoneum. We propose to consider whether he is justified in so doing, confining our attention to tubercular disease.

In tubercular disease of the peritoneum we may have ascites. The ascites may be unaccompanied by symptoms, or we may have a hectic temperature, pain in the abdomen, and perhaps some vomiting and diarrhœa. In these cases we should always carefully explore the abdomen for bands and lumps, dipping with the finger through the ascitic fluid as it were. These bands and lumps are due to thickening of the omentum, and matting together of the of gut.

But we may have no ascites at all, only the thickening and matting together; or sometimes the disease begins with ascites, and then the fluid gets absorbed, and we find much matting together and thickening previously obscured by the fluid. Sometimes in the dry form friction can be detected by the hand. Bacilli have not, we believe, been found in the fluid though they have in the tubercular growth in these cases. Hence as in all such cases their absence is of no diagnostic value.

If a child presents ascites without any other symptom, as they sometimes do, the great probability is that it is suffering from tubercular disease of the peritoneum.

But we must not go at length into the symptoms, though we ought to be familiar with any disease we are called on to treat surgically, and it is even more important that we should understand its pathology.

In tubercular disease of the peritoneum we have tubercles and tubercular masses, scattered over the membrane in all directions, and causing great thickening, and penitoneal inflamation which produces adhesion of neighbouring coils of bowel. Sometimes the omentum gets drawn together into a cord-like structure. Often the adherent intestines are tucked away under a greatly thickened omentum, so that on opening the abdomen only a large mass is seen in which no gut can be recognised. This is very important, as surgically it might be misleading. The other day we made a post-mortem and found such a mass looking more like a solid growth than a mass of bowel. In this case the peritoneum was full of liquid fæcal matter, which had escaped from two openings into the bowel; one large enough to admit the little finger, with eversion of the mucous coat, which must have existed some considerable time; the other, a small perforation just admitting a full-sized catheter, with thinned edge, opening into another coil of small intestine. It is a very interesting question how these openings were formed. There were no traces of ulceration, active or healed, in the bowel at the spot perforated or near; nor were ulcers found elsewhere in the bowel in this case, though they are so often associated with tubercular peritonitis. Dr. West (in his classical work on Children's Diseases) says: "Perforation of the intestines, too, is produced in these cases from without inwards, not by destruction of the coats of the bowel by tubercular ulceration in its interior." Probably this perforation takes place from absorption of tubercular deposits in the wall of the bowel.

There is another very important point in connection with the surgery of tubercular peritonitis illustrated by the same case, and that is the close resemblance of much of the tubercular growth to little masses of fat. At first we were in doubt if these were not fat, but they grew on the peritoneum of the liver, as well as on the mesentery. They were more friable than fat.

It is remarkable how little the mesenteric glands seem to suffer in some of these cases. In the case already alluded to no enlarged mesenteric glands could be discovered, though there may have been some hidden away amongst adherent bowel, but certainly they were not much enlarged, whereas the bronchial glands were markedly caseous, although there were only two minute tubercles in the lungs. Tuber cular enlargement of these glands used to be considered a very important condition. Now we know that they seldom exceed the size of a walnut when tubercular, and are very rarely felt during life. Probably the thickenings of tubercular peritonitis have been mistaken for them, and the very large glandular growths have been lymphomatous in character. These glands are certainly often enlarged in tubercular peritonitis, but are not usually felt.

Sometimes a fæcal fistula forms in the course of this disease. The bowel becomes matted together and adherent to the abdominal wall; perforation occurs, an abscess forms, shut in by the adhesions, and pus followed by fæcal matter escapes at the umbilicus. We have lately seen such a case. The firm induration in the abdomen around the openings was largely removed by gently scraping out the suppurating cavity, but the fæcal discharge persisted when the patient left the hospital, though her general health was good.

And now what can we do for these cases surgically? First we must remember that tubercular peritonitis is not by any means a uniformly fatal disease. Many cases with and without ascites recover. Does surgical treatment give any better results than non-operative treatment on the natural course of the disease?

It is claimed for laparotomy in the ascitic form, that it cures when tapping alone would not; and that it does not much matter whether we merely open the cavity and thoroughly evacuate the fluid or wash out with sterilised hot water or drain, the result is said to be equally successful. In Germany they even do abdominal section when there is no ascites.

How laparotomy can do good in this disease it is difficult to see. That the removal of the fluid hinders the growth of the bacillus is maintained by some. They say the ascitic fluid acts as a culture fluid. But surely the bacillus can thrive in the peritoneum without any "culture fluid," and the removal of the fluid will hardly place it under unfavourable conditions, seeing that it is already in living tissue. It is indeed difficult to understand how removal of the fluid by laparotomy can have any more effect on the tubercles than tapping, although it may be a complete removal. Possibly the more thorough removal allows adhesions to form, obliterating the peritoneal cavity to so large an extent that ascites cannot again become a prominent feature. In the dry form of the disease, how laparotomy can do good seems quite inexplicable. It would be impossible to get away the tubercles scattered amongst adherent masses of intestine, and intimately connected with the peritoneal coat; besides, to attempt this would require an enormous incision, for we should have to see, as well as feel, what we were about. It seems to us that, *a priori*, there can be no chance of doing good by laparotomy in these cases, unless localised collections of pus are present between the adherent coils, and occasionally they are, but we cannot diagnose them. Are we, then, to open the abdomen on the chance of finding them in a disease of fair prognosis?

In cases where localised dulness and fluctuation (true fluctuation, not the semi-fluctuation of a mass of adherent bowel) are present, indicating the probable existence of a localised collection of pus, laparotomy, flushing, and drainage are clearly called for.

In order to prove the utility of surgical interference, it must be shown not only that so many cases recover, but how many more recover of the same kind than when left alone. We read of "a case of tubercular peritonitis cured by laparotomy," and perhaps we might be disposed to regard this as a cure only thus to be brought about. But it is not so. Many cases recover if left alone. Of course, if a case has been tapped, and is steadily going from bad to worse, and then quickly recovers under laparotomy, we have distinct evidence in favour of it, but we must not think that every case that recovers after laparotomy would not have recovered without. If we are to be guided by statistics at all we must first have reliable ones of recovery without operation. The only ones of this kind that we can find are some given by Dr. Finlay.* Out of 35 cases in the Middlesex Hospital, 19 were discharged as recovered or relieved. This seems to us too indefinite to be of much value, but Dr. Finlay's statement "that recovery is much more frequent than is usually thought" is echoed on all sides.

Prof. König at the last Berlin Medical Congress referred to 130 cases, out of which 84 or 65 per cent. were cured. He operates in cases without ascites, and he removes solid masses with the sharp spoon, scissors, or knife. More cases got well without antiseptic washing out than with. Kussmaul at a previous congress of German surgeons brought forward 30 cases operated on by himself with only five deaths. M. Maurange recommends tapping, then antiseptic flushing, and injection of a mixture of four grammes of iodoform in 100 of liquid oil of vaselin. He says he has seen good results follow, and there is no danger of poisoning with so weak a solution and so diseased a condition of the peritoneum.

Most of the early cases operated on in this country, and we believe also in Germany, were done under a misapprehension as to their nature—they were thought to be ovarian cysts (as in Sir Spencer Wells' classical case), so closely does the ascitic form of tubercular peritonitis resemble this disease. We have lately seen a case in which no one could have confidently diagnosed tubercular peritonitis rather than ovarian cyst, though she was suffering from the former disease. A hectic of 100 at night was the only symptom in favour of tubercular peritonitis, and the physical signs much more resembled ovarian cyst. The intestines were bound down in the flanks. The disease occurred in a young woman, and was of a very latent form. Happily in these doubtful cases laparotomy is the right treatment in the one case, and certainly if not shown to be absolutely necessary, is recognised as a very favourable method of treatment in the other. Possibly the existence of signs of tubercular disease in the lungs or intestines may sometimes help in our difficult diagnosis. Can we be guided by examination of the fluid withdrawn by an exploring needle? Unfortunately not. Bland Sutton in his recent book on surgical diseases of the ovaries, says there are really no characters, microscopic, chemical, or spectroscopic, which can enable us to decide between ascites and the fluid in an ovarian cyst. In the case Mr. Lawford Knaggs operated on in 1887, in which he diagnosed an ovarian cyst, paralbumen was present in the fluid, a chemical substance, stated in text books only to be present in ovarian cysts.

* *British Medical Journal*, 90, Vol. 2, pages 10, 11.

